# Amphetamine Derivatives as Monoamine Oxidase Inhibitors

**DOI:** 10.3389/fphar.2019.01590

**Published:** 2020-01-23

**Authors:** Miguel Reyes-Parada, Patricio Iturriaga-Vasquez, Bruce K. Cassels

**Affiliations:** ^1^ Centro de Investigación Biomédica y Aplicada (CIBAP), Escuela de Medicina, Facultad de Ciencias Médicas, Universidad de Santiago de Chile, Santiago, Chile; ^2^ Facultad de Ciencias de la Salud, Universidad Autónoma de Chile, Talca, Chile; ^3^ Departamento de Ciencias Químicas y Recursos Naturales, Facultad de Ingeniería y Ciencias, Universidad de la Frontera, Temuco, Chile; ^4^ Departamento de Química, Facultad de Ciencias, Universidad de Chile, Santiago, Chile

**Keywords:** monoamine oxidase, amphetamine derivatives, serotonin syndrome, serotonin transporter, dopamine transporter, norepinephrine transporter, monoamine oxidase-A

## Abstract

Amphetamine and its derivatives exhibit a wide range of pharmacological activities, including psychostimulant, hallucinogenic, entactogenic, anorectic, or antidepressant effects. The mechanisms of action underlying these effects are usually related to the ability of the different amphetamines to interact with diverse monoamine transporters or receptors. Moreover, many of these compounds are also potent and selective monoamine oxidase inhibitors. In the present work, we review how structural modifications on the aromatic ring, the amino group and/or the aliphatic side chain of the parent scaffold, modulate the enzyme inhibitory properties of hundreds of amphetamine derivatives. Furthermore, we discuss how monoamine oxidase inhibition might influence the pharmacology of these compounds.

## Introduction

**Figure 1:**
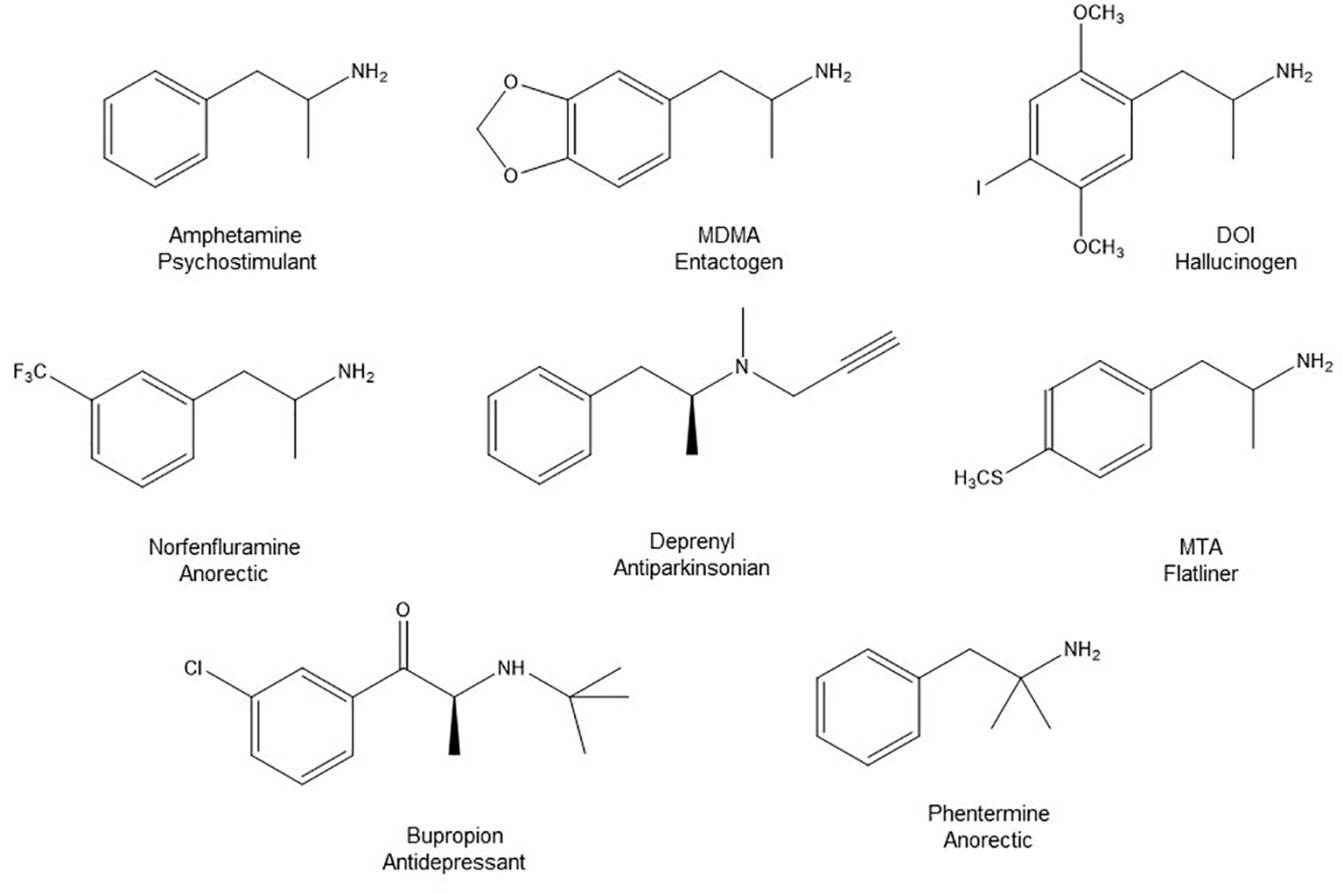
Chemical structures of some AMPH derivatives.

Since its first description as “Phenisopropylamin” more than a century ago (Edeleano, 1887), amphetamine (1-phenylpropan-2-amine, phenylisopropylamine, amfetamine, alpha-methylphenethylamine; AMPH; **Figure 1**) has received considerable attention due to its multiple psychotropic effects, first noted in the early 1930s (Prinzmetal and Bloomberg, 1935). Nowadays, AMPH is indicated for the treatment of attention deficit hyperactivity disorder (Heal et al., 2013), narcolepsy (Dauvilliers and Barateau, 2017), and— in the form of its prodrug lisdexamfetamine— binge-eating disorder (Hilbert, 2019). Its psychostimulant effects are usually related to its catecholamine-releasing properties, which arise from its ability to compete with dopamine (DA) and norepinephrine (NE) for uptake into the nerve terminals, and to induce reverse transport *via* the corresponding transporter (DAT and NET, respectively). Nevertheless, its polypharmacological profile involves actions upon other monoaminergic targets such as the serotonin (5-HT) transporter (SERT), the vesicular monoamine transporter and monoamine oxidase (MAO) (Sulzer et al., 2005; Heal et al., 2013).

A remarkable characteristic of AMPH is that subtle structural variations can produce drastic changes in its pharmacodynamics, and lead to compounds that interact differentially with several biogenic amine target proteins. Consequently, the AMPH skeleton has served as a privileged scaffold for the design and synthesis of hundreds of derivatives with many different and often useful activities, but also conveying misuse potential (Biel and Bopp, 1978; Nichols, 1994; Glennon, 1999; Rothman and Baumann, 2003; Welter-Luedeke and Maurer, 2016). Thus, the diversity of mechanisms of action of AMPH derivatives determines a many-colored palette of pharmacological activities in humans, including psychostimulant, entactogenic, psychedelic, anorectic, nootropic, and antidepressant effects. It is noteworthy that the structural changes also modify toxicological properties and abuse liability of AMPH derivatives (Fleckenstein et al., 2007; Rothman et al., 2007; Simmler et al., 2013; Barbosa et al., 2015).

**Figure 2:**
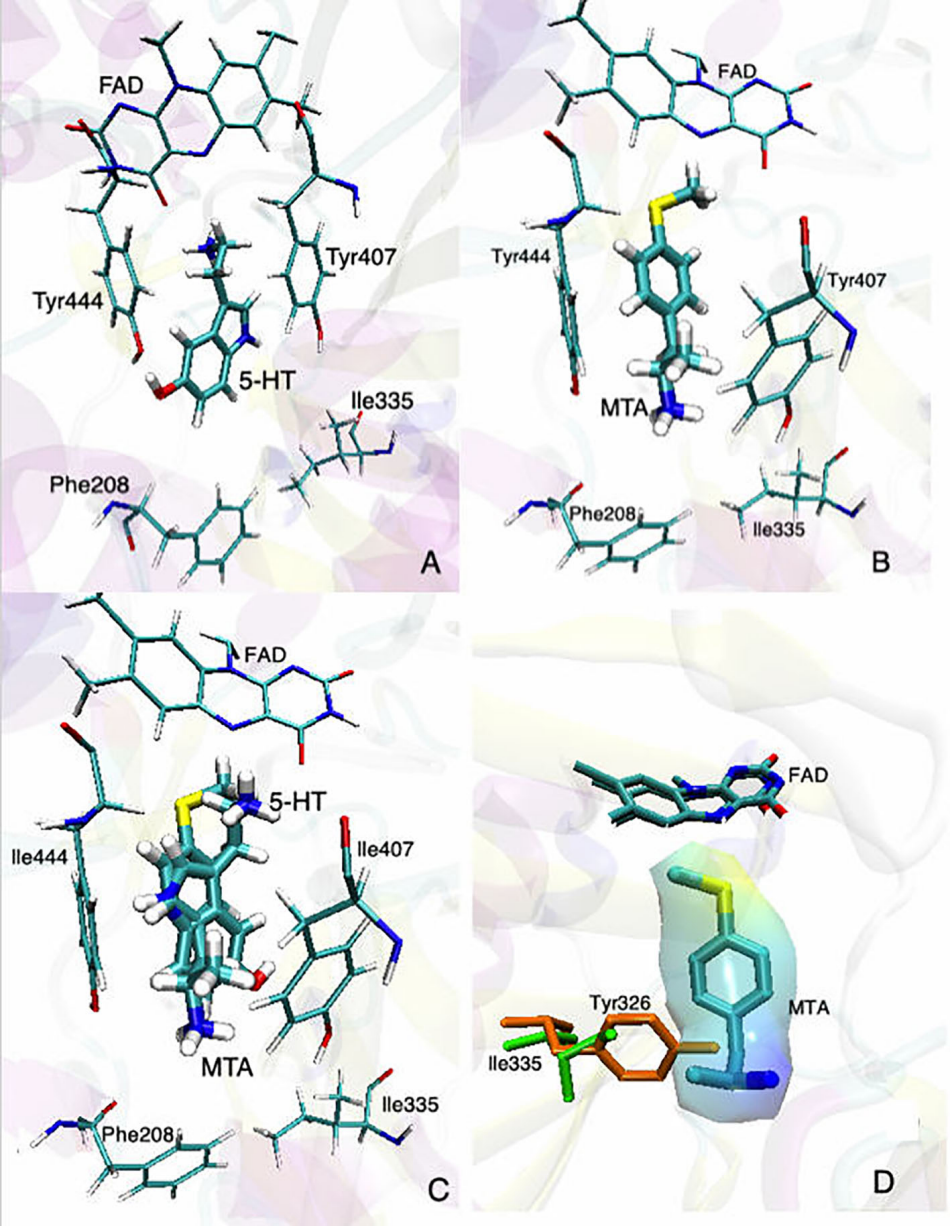
Binding modes of **(A)** 5-HT and **(B)** MTA to MAO-A (PDB: 2BXS). **(C)** Superimposed structures of 5-HT and MTA docked into the active site of MAO-A. **(D)** Superimposed binding sites of MAO-A (green residues) and MAO-B (orange residues) with MTA already docked into the active site of MAO-A; the “wrapper” around MTA represents the solvent accessible surface area (SASA). In all cases, for the sake of clarity, only the most relevant residues are shown. Docking conditions were as in Fierro et al., 2007.

MAO (monoamine oxygen oxidoreductase (deaminating) (flavin-containing); EC 1.4.3.4) is the main catabolic enzyme for biogenic monoamines such as NE, DA, 5-HT, and β-phenethylamine, and also for dietary and xenobiotic amines such as tyramine and benzylamine. MAO exists in two isoforms termed MAO-A and MAO-B. Both isozymes are outer mitochondrial membrane-bound flavoproteins, with the FAD cofactor covalently bound to the enzyme. The metabolic reaction involves the generation of an imine intermediate and the reduction of the flavin cofactor, which is reoxidized by molecular oxygen producing hydrogen peroxide. The imine intermediate is hydrolyzed, in a non-enzymatic process, generating ammonia and the corresponding aldehyde (Shih et al., 1999; Tipton et al., 2004; Edmondson et al., 2007). Although both isoforms have similar catalytic activities, they differ in their molecular genetics, physiological roles, tissue distribution, substrate preference, and inhibitor selectivity (Reyes-Parada et al., 2005). In the central nervous system, catecholaminergic neurons contain predominantly MAO-A, whereas serotonergic neurons express MAO-B (Westlund et al., 1988; Luque et al., 1995). MAO-A preferentially metabolizes 5-HT and is irreversibly inhibited by nanomolar concentrations of clorgyline, whereas MAO-B preferentially catalyzes the oxidative deamination of phenethylamine and benzylamine and is irreversibly inhibited by nanomolar concentrations of *l*-deprenyl. DA and NE are non-selective substrates of both isoforms (Youdim et al., 2006). MAO inhibitors (MAOI) are currently used in the treatment of diverse neuropsychiatric and neurological disorders, including depression and Parkinson's disease (Cesura and Pletscher, 1992; Youdim et al., 2006; Finberg and Rabey, 2016; Kumar et al., 2017). In 2002, Binda and colleagues (Binda et al., 2002) published a seminal article showing the high-resolution structure of human MAO-B. Subsequent structures of this enzyme (Binda et al., 2003; Binda et al., 2004), as well as that of rat (Ma et al., 2004) and human MAO-A (De Colibus et al., 2005; Son et al., 2008), have allowed detailed comparison of the overall structures of both isoforms and their active sites (Binda et al., 2011; Iacovino et al., 2018). Thus, the substrate/inhibitor binding site of both isozymes (see **Figure 2**) can be described as a pocket lined by the isoalloxazine ring and several aliphatic and aromatic residues. A critical role of Y444, Y407, G215, and I180 of MAO-A (Y435, Y398, G206, and L171 being the corresponding residues in MAO-B) in the orientation and stabilization of the substrate/inhibitor binding can be inferred from the X-ray diffraction data. The availability of MAO crystal structures has allowed a quicker pace in the rational design of novel MAOIs and in the understanding of catalytic and inhibitory mechanisms. Thus, a vast number of studies in which molecular simulation approaches have been used to rationalize and/or to predict the functional interactions between the proteins and their substrates or inhibitors have been reported recently (Ferino et al., 2012; Vianello et al., 2016; Dhiman et al., 2017; Dhiman et al., 2018).

In the following pages, we review the effects of several dozen AMPH derivatives upon MAOs and describe, through the analysis of a set of representative examples, how structural modifications on the aromatic ring, the amino group and/or the aliphatic side chain of the parent scaffold, modulate the enzyme inhibitory properties of this type of compounds.

## General Nature of Mao Inhibition by Amph Derivatives

Since the first pharmacological study more than 50 years ago proving the existence of the two enzyme isoforms (Johnston, 1968), hundreds of AMPH derivatives have been tested as MAOIs. **Tables 1**–**5** summarize the effects of a subset of these compounds. It should be noted that, as expected for results obtained from different laboratories over a long period of time, the methodological approaches used to assess MAO inhibition are diverse. Thus, a variety of biochemical assays to follow MAO activities (e.g. radiometric, luminometric, spectrophotometric, electrochemical, fluorometric), substrates (e.g. 5-HT, kynuramine, β-phenethylamine, benzylamine, 4-dimethylaminophenethylamine), inhibition parameters (e.g. IC_50_, K_i_), tissue sources (e.g. brain, lung, liver), and species (e.g. rat, mouse, human from fresh tissue, human recombinant heterologously expressed in yeast), have been employed in these experiments. Therefore, although we made an effort to consider results obtained under relatively similar conditions for comparative analysis, the reader should bear in mind this limitation when evaluating the data presented below.

**Table 1:** MAO inhibitory activity of AMPH derivatives and amiflamine analogues.

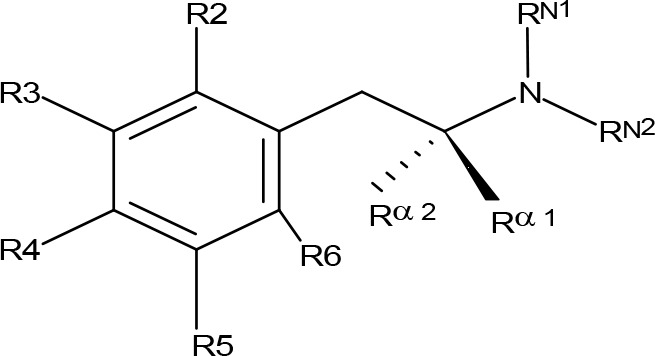											
										MAOI Activity IC_50_(Ki)^a^ (µM)	
Compound^b^	R2	R3	R4	R5	R6	R^α1^	R^α2^	R^N1^	R^N2^	MAO-A	MAO-B
(+)-Amphetamine	H	H	H	H	H	CH_3_	H	H	H	20.0^c^;4.9^d^;33.8^e^	770^c^;118^d^;161^e^
Amphetamine	H	H	H	H	H	HCH_3_		H	H	11.0^f^(5.3^g^)	236^g^
(-)-Amphetamine	H	H	H	H	H	H	CH_3_	H	H	70.0^c^;203^e^	600^c^;180^e^
Methamphetamine	H	H	H	H	H	HCH_3_		CH_3_	H	41^h^(17.2^g^)	> 200^h^(297)^g^
Phentermine	H	H	H	H	H	CH_3_	CH_3_	H	H	143^i^(88^d^;196^g^)	285^i^(310^d^;138^g^)
AEPEA	H	H	H	H	H	HCH_2_CH_3_		H	H	14.0^g^	234^g^
*N*,α-DEPEA	H	H	H	H	H	HCH_2_CH_3_		CH_2_CH_3_	H	251^g^	159^g^
Amiflamine/(+)-FLA336	CH_3_	H	N(CH_3_)_2_	H	H	CH_3_	H	H	H	0.8^j^;2.0^f^	> 1000^j^
FLA336	CH_3_	H	N(CH_3_)_2_	H	H	HCH_3_		H	H	2.7^k^	440^k^
(-)-FLA336	CH_3_	H	N(CH_3_)_2_	H	H	H	CH_3_	H	H	3.0^j^	125^l^
FLA289	H	H	N(CH_3_)_2_	H	H	HCH_3_		H	H	3.7^l^;2.0^m^	400^l^
FLA727	H	H	NHCH_3_	H	H	HCH_3_		H	H	0.55^l^-1.2^m^	1500^l^
(+)-FLA788	CH_3_	H	NHCH_3_	H	H	CH_3_	H	H	H	0.13^j^	> 1000^j^
FLA558	F	H	N(CH_3_)_2_	H	H	HCH_3_		H	H	1.2^k^	120^k^
FLA314	Cl	H	N(CH_3_)_2_	H	H	HCH_3_		H	H	0.21^k^	80^k^
FLA405	Br	H	N(CH_3_)_2_	H	H	HCH_3_		H	H	0.22^k^	100^k^
FLA365	Cl	H	N(CH_3_)_2_	H	Cl	HCH_3_		H	H	0.013^l^	180^l^
FLA450	Cl	H	N(CH_3_)_2_	H	H	HCH_2_CH_3_		H	H	0.38^k^	75^k^
FLA463	Cl	H	N(CH_3_)_2_	H	H	CH_3_	CH_3_	H	H	1.2^k^	700^k^
FLA717	CH_3_	H	N(CH_3_)_2_	H	H	CH_3_	CH_3_	H	H	12.0^k^	2100^k^
FLA384	H	CH_3_	N(CH_3_)_2_	H	H	HCH_3_		H	H	8.0^l^	650^l^
(+)NBF003	CH_3_	H	N(CH_3_)_2_	Br	H	CH_3_	H	H	H	1.1^l^	480^l^

^a^IC_50_ and/or Ki are reported, depending on the reference considered. ^b^Chemical name and/or common acronym and/or common name is given. When not indicated, the compound is the racemic mixture. ^c^ Mantle et al., 1976. ^d^ Ulus et al., 2000. ^e^ Robinson, 1985. ^f^ Scorza et al., 1997.^g^ Santillo, 2014. ^h^ Matsumoto et al., 2014. ^i^ Kilpatrick et al., 2001. ^j^ Ask et al., 1982b. ^k^ Ask et al., 1982a. ^l^ Ask et al., 1985. ^m^ Reyes-Parada et al., 1994a.

**Table 2:** MAO inhibitory activity of AMPH derivatives monosubstituted in the aromatic ring.

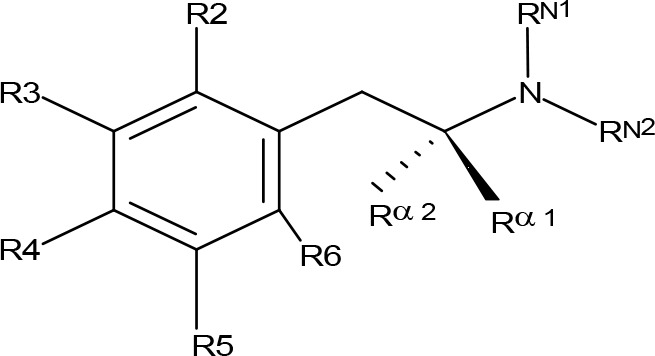											
										MAOI Activity IC_50_(Ki)^a^ (µM)	
Compound^b^	R2	R3	R4	R5	R6	R^α1^	R^α2^	R^N1^	R^N2^	MAO-A	MAO-B
PMA/4-MeOA	H	H	OCH_3_	H	H	HCH_3_		H	H	0.3^c^;0.6^d^(0.2^e^)	45^d^(530^e^)
2-MeOA	OCH_3_	H	H	H	H	HCH_3_		H	H	9.0^e^	350^e^
3-MeOA	H	OCH_3_	H	H	H	HCH_3_		H	H	23^e^	1940^e^
PMMA	H	H	OCH_3_	H	H	HCH_3_		CH_3_	H	1.7^d^	58^d^
4-EtOA	H	H	OCH_2_CH_3_	H	H	HCH_3_		H	H	0.22^f^	> 100^f^
4-PrOA	H	H	O(CH_2_)_2_CH_3_	H	H	HCH_3_		H	H	0.13^f^	> 100^f^
4-BuOA	H	H	O(CH_2_)_3_CH_3_	H	H	HCH_3_		H	H	0.32^f^	> 100^f^
4-BzOA	H	H	OCH_2_Phe	H	H	HCH_3_		H	H	3.42^f^	0.71^f^
MTA	H	H	SCH_3_	H	H	HCH_3_		H	H	0.25^g^	NE^g^
(+)-MTA	H	H	SCH_3_	H	H	CH_3_	H	H	H	0.13^h^	NE^h^
(-)-MTA	H	H	SCH_3_	H	H	H	CH_3_	H	H	2.04^g^	NE^g^
NMMTA	H	H	SCH_3_	H	H	HCH_3_		CH_3_	H	0.89^g^	NE^g^
DMMTA	H	H	SCH_3_	H	H	HCH_3_		CH_3_	CH_3_	2.10^g^	NE^g^
NEMTA	H	H	SCH_3_	H	H	HCH_3_		CH_2_CH_3_	H	1.80^g^	NE^g^
DEMTA	H	H	SCH_3_	H	H	HCH_3_		CH_2_CH_3_	CH_2_CH_3_	6.45^g^	NE^g^
NPMTA	H	H	SCH_3_	H	H	HCH_3_		(CH_2_)_2_CH_3_	H	2.41^g^	> 10^g^
DPMTA	H	H	SCH_3_	H	H	HCH_3_		(CH_2_)_2_CH_3_	(CH_2_)_2_CH_3_	> 10^g^	NE^g^
NBzMTA	H	H	SCH_3_	H	H	HCH_3_		CH_2_Phe	H	> 100^f^	> 100^f^
MTAB	H	H	SCH_3_	H	H	HCH_2_CH_3_		H	H	0.84^g^	NE^g^
ETA	H	H	SCH_2_CH_3_	H	H	HCH_2_CH_3_		H	H	0.10^c^	29^c^
(+)-ETA	H	H	SCH_2_CH_3_	H	H	CH_3_	H	H	H	0.075^h^	> 100^h^
(+)-PTA	H	H	S(CH_2_)_2_CH_3_	H	H	CH_3_	H	H	H	0.030^h^	14.0^h^
ITA	H	H	SCH(CH_3_)_2_	H	H	HCH_3_		H	H	0.40^c^	8.1^c^
(+)-BTA	H	H	S(CH_2_)_3_CH_3_	H	H	HCH_3_		H	H	0.022^h^	4.6^h^
MSOA	H	H	SOCH_3_	H	H	HCH_3_		H	H	> 100^i^	NT
MSO2A	H	H	SO_2_CH_3_	H	H	HCH_3_		H	H	> 100^i^	NT
PCA/*p*-Chloroamphetamine	H	H	Cl	H	H	HCH_3_		H	H	4.0^c^;1.9^j^	NE^c^
PBA/*p*-Bromoamphetamine	H	H	Br	H	H	HCH_3_		H	H	1.5^j^	NT
PFA/*p*-Fluoroamphetamine	H	H	F	H	H	HCH_3_		H	H	16^j^	NT
POHA	H	H	OH	H	H	HCH_3_		H	H	24.0^k^	NE^k^
(+)-Fenfluramine	H	CF_3_	H	H	H	CH_3_	H	CH_2_CH_3_	H	256^l^	800^l^
Fenfluramine	H	CF_3_	H	H	H	HCH_3_		CH_2_CH_3_	H	440^m^	720^m^
(-)-Fenfluramine	H	CF_3_	H	H	H	H	CH_3_	CH_2_CH_3_	H	115^l^	685^l^
(+)-Norfenfluramine	H	CF_3_	H	H	H	CH_3_	H	H	H	36^l^	160^l^

^a^IC_50_ and/or Ki are reported, depending on the reference considered. ^b^Chemical name and/or common acronym and/or common name is given. When not indicated the compound is the racemic mixture. ^c^ Scorza et al., 1997. ^d^ Matsumoto et al., 2014. ^e^ Green and el Hait, 1980. ^f^ Vilches-Herrera et al., 2009. ^g^ Hurtado-Guzmán et al., 2003. ^h^ Fierro et al., 2007. ^i^ Vallejos et al., 2002. ^j^ Fuller et al., 1975. ^k^ Arai et al., 1990. ^l^ Kilpatrick et al., 2001. ^m^ Leonardi and Azmitia, 1994. NE, No effect at 100 µM; NT, Not tested.

**Table 3:** MAO inhibitory activity of β-substituted AMPH derivatives.

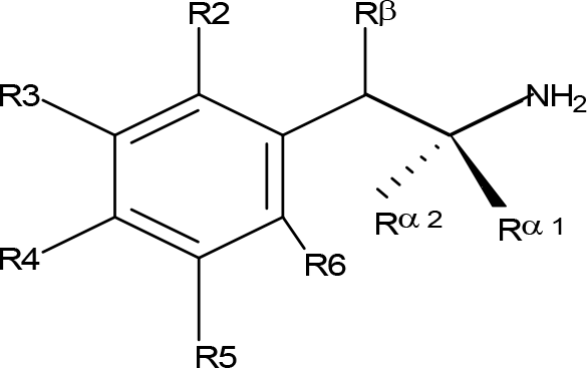										
									MAOI Activity IC_50_^a^ (µM)	
Compound^b^	R^β^	R2	R3	R4	R5	R6	R^α1^	R^α2^	MAO-A	MAO-B
Cathinone	=O	H	H	H	H	H	HCH_3_		NE	NE
4-MetOCat	=O	H	H	OCH_3_	H	H	HCH_3_		77.0	NE
4-EtOCat	=O	H	H	OCH_2_CH_3_	H	H	HCH_3_		37.0	> 100
4-PropOCat	=O	H	H	O(CH_2_)_2_CH_3_	H	H	HCH_3_		7.2	8.9
4-ButOCat	=O	H	H	O(CH_2_)_3_CH_3_	H	H	HCH_3_		14.4	6.0
(+)4-ButOCat	=O	H	H	O(CH_2_)_3_CH_3_	H	H	CH_3_	H	29.5	5.6
(-)4-ButOCat	=O	H	H	O(CH_2_)_3_CH_3_	H	H	H	CH_3_	6.8	6.4
4-MetSCat	=O	H	H	SCH_3_	H	H	HCH_3_		45.0	> 100
(+)4-MetSCat	=O	H	H	SCH_3_	H	H	CH_3_	H	44.5	> 100
(-)4-MetSCat	=O	H	H	SCH_3_	H	H	H	CH_3_	38.9	NT
4-EtSCat	=O	H	H	S CH_2_CH_3_	H	H	HCH_3_		15.1	> 100
(+)4-EtSCat	=O	H	H	S CH_2_CH_3_	H	H	CH_3_	H	12.9	> 100
(-)4-EtSCat	=O	H	H	S CH_2_CH_3_	H	H	H	CH_3_	38.0	NT
4-MetONEPhe	OH	H	H	OCH_3_	H	H	HCH_3_		9.8	NE
4-EtONEPhe	OH	H	H	O CH_2_CH_3_	H	H	HCH_3_		7.0	NE
4-PropONEPhe	OH	H	H	O (CH_2_)_2_CH_3_	H	H	HCH_3_		2.8	100
4-ButONEPhe	OH	H	H	O (CH_2_)_3_CH_3_	H	H	HCH_3_		4.7	65
4-OHNEPhe	OH	H	H	OH	H	H	HCH_3_		220.0^c^	NE^c^
4-MetSNEPhe	OH	H	H	SCH_3_	H	H	HCH_3_		7.3	NE
4-EtSNEPhe	OH	H	H	S CH_2_CH_3_	H	H	HCH_3_		1.9	> 100
4-PropSNEPhe	OH	H	H	S (CH_2_)_2_CH_3_	H	H	HCH_3_		1.7	> 100
BMetOA	OCH_3_	H	H	H	H	H	HCH_3_		> 100	> 100
B,4DMetOA	OCH_3_	H	H	OCH_3_	H	H	HCH_3_		77.5	> 100
BMetSA	OCH_3_	H	H	SCH_3_	H	H	HCH_3_		50.6	> 100

^a^Unless stated, IC_50_ values are from Osorio-Olivares et al., 2004. ^b^Chemical name and/or common acronym and/or common name is given. When not indicated the compound is the racemic mixture. ^c^ Arai et al., 1990. NE, No effect at 100 µM; NT, Not tested.

**Table 4:** MAO inhibitory activity of AMPH derivatives polysubstituted in the aromatic ring.

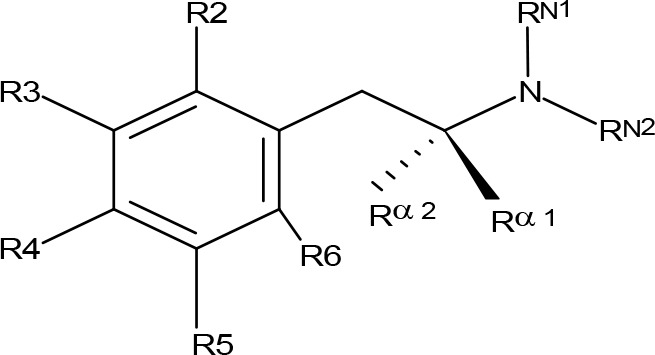												
											MAOI Activity IC_50_(Ki)^a^ (µM)	
Compound^b^	R2	R3	R4	R5	R6	R^α^^1^	R^α^^2^		R^N1^	R^N2^	MAO-A	MAO-B
2,4-DMA	OCH_3_	H	OCH_3_	H	H	HCH_3_			H	H	0.6^c^	NE^c^
3,4-DMA	H	OCH_3_	OCH_3_	H	H	HCH_3_			H	H	20^c^	NE^c^
2,5-DMA	OCH_3_	H	H	OCH_3_	H	HCH_3_			H	H	> 100^f^	NE^f^
3,4,5-TMA	H	OCH_3_	OCH_3_	OCH_3_	H	HCH_3_			H	H	NE^c^;NI^d^	NE^c^;NI^d^
2,4,5-TMA	OCH_3_	H	OCH_3_	OCH_3_	H	HCH_3_			H	H	NE^c^	NE^c^
2,4,6-TMA	OCH_3_	H	OCH_3_	H	OCH_3_	HCH_3_			H	H	0.4^e^	NE^e^
2-Br-DMA	Br	H	OCH_3_	OCH_3_	H	HCH_3_			H	H	9.3^c^	NE^c^
5-Br-DMA	OCH_3_	H	OCH_3_	Br	H	HCH_3_			H	H	13.0^c^	NE^c^
2-NO_2_-DMA	NO_2_	H	OCH_3_	OCH_3_	H	HCH_3_			H	H	NE^c^	NE^c^
6-Cl-DMA	OCH_3_	H	OCH_3_	H	Cl	HCH_3_			H	H	0.07^e^	NE^e^
ALEPH-1	OCH_3_	H	SCH_3_	OCH_3_	OCH_3_	HCH_3_			H	H	5.1^c^	NE^c^
ALEPH-2	OCH_3_	H	SCH_2_CH_3_	OCH_3_	H	HCH_3_			H	H	3.2^c^	NE^c^
4-PrS-DMA	OCH_3_	H	S(CH_2_)_2_CH_3_	OCH_3_	H	HCH_3_			H	H	2.4^e^	NE^e^
4-BuS-DMA	OCH_3_	H	S(CH_2_)_3_CH_3_	OCH_3_	H	HCH_3_			H	H	2.9^e^	NE^e^
4-PentS-DMA	OCH_3_	H	S(CH_2_)_4_CH_3_	OCH_3_	H	HCH_3_			H	H	14.3^e^	NE^e^
2,5-DM-MTAB	OCH_3_	H	SCH_3_	OCH_3_	H	HCH_2_CH_3_			H	H	30.9^e^	NE^e^
2,5-DM-ETAB	OCH_3_	H	SCH_2_CH_3_	OCH_3_	H	HCH_2_CH_3_			H	H	11.8^e^	NE^e^
2,6-DM-MTA	OCH_3_	H	SCH_3_	H	OCH_3_	HCH_3_			H	H	0.30^e^	NE^e^
2,6-DM-ETA	OCH_3_	H	SCH_2_CH_3_	H	OCH_3_	HCH_3_			H	H	0.08^e^	NE^e^
4-ESO-2,5-DMA	OCH_3_	H	SOCH_3_	OCH_3_	H	HCH_3_			H	H	> 100^f^	NT
4-ESO2-2,5-DMA	OCH_3_	H	SO_2_CH_3_	OCH_3_	H	HCH_3_			H	H	NE^f^	NT
DOM	OCH_3_	H	CH_3_	OCH_3_	H	HCH_3_			H	H	24.0^c^	NE^c^
DOI	OCH_3_	H	I	OCH_3_	H	HCH_3_			H	H	24^c^;37^d^	NE^c^
DOB	OCH_3_	H	Br	OCH_3_	H	HCH_3_			H	H	100^c^	NE^c^
DON	OCH_3_	H	NO_2_	OCH_3_	H	HCH_3_			H	H	NE^c^	NE^c^
DOTFM	OCH_3_	H	CF_3_	OCH_3_	H	HCH_3_			H	H	NE^c^	NE^c^
MDA	H	CH_2_-O-CH_2_		H	H	HCH_3_			H	H	9.3^c^(8.5^g^)	NE^c^
2Br-MDA	Br	H	CH_2_-O-CH_2_		H	HCH_3_			H	H	13.0^c^	64.0^c^
2Cl-MDA	Cl	H	CH_2_-O-CH_2_		H	HCH_3_			H	H	6.3^c^	38.0^c^
2NO_2_-MDA	NO_2_	H	CH_2_-O-CH_2_		H	HCH_3_			H	H	NE^c^	NE^c^
MDMA	H	CH_2_-O-CH_2_		H	H	HCH_3_			CH_3_	H	30^c^(24.7^g^)	NE^c^
(+)-MDMA	H	CH_2_-O-CH_2_		H	H	CH_3_		H	CH_3_	H	44^h^(22^h^)	370^h^
(-)-MDMA	H	CH_2_-O-CH_2_		H	H	H		CH_3_	CH_3_	H	56^h^(28.3^h^)	378^h^

^a^IC_50_ and/or Ki are reported, depending on the reference considered. ^b^Chemical name and/or common acronym and/or common name is given. When not indicated the compound is the racemic mixture. ^c^ Scorza et al., 1997. ^d^ Matsumoto et al., 2014. ^e^ Gallardo-Godoy et al., 2005. ^f^ Vallejos et al., 2002. ^g^ Steuer et al., 2016. ^h^ Leonardi and Azmitia, 1994. NE, No effect at 100 µM; NI, No inhibition at 200 µM; NT, Not tested.

**Table 5:** MAO inhibitory activity of some AMPH derivatives containing aromatic systems larger than benzene.

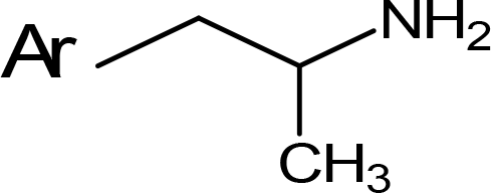			
		MAOI Activity IC_50_(Ki)^a^ (µM)	
Compound^b^	Ar	MAO-A	MAO-B
NIPA/PAL-287	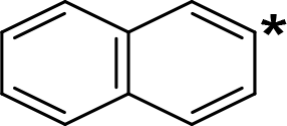	0.42^c^	> 100^c^
6-MeO-NIPA	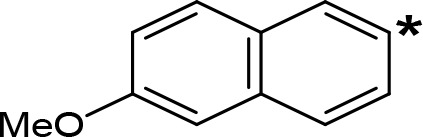	0.18^c^	16.3^c^
6-EtO-NIPA	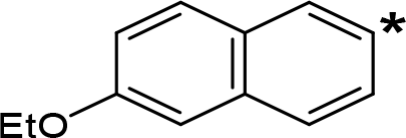	0.45^c^	13.6^c^
6-PrO-NIPA	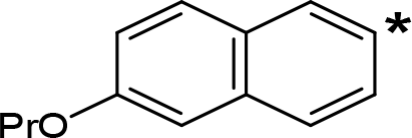	0.68^c^	13.5^c^
6-BuO-NIPA	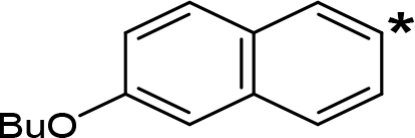	1.53^c^	NT
6-MeS-NIPA	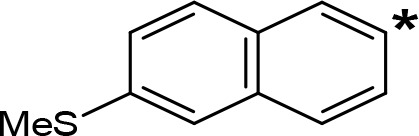	0.50^c^	NT
2-Benzofuryl-IPA	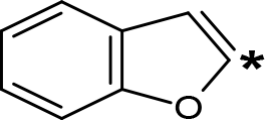	0.80^d^	> 100^d^
AMT	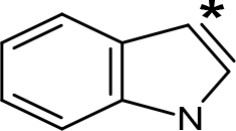	0.38^e^	> 10^e^
4-MeO-AMT	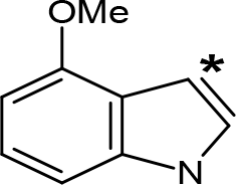	1.4^e^	> 10^e^
5-MeO-AMT	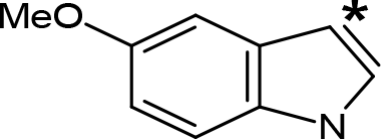	31^e^	> 10^e^
5-Me-AMT	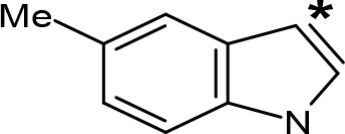	1.5^e^	> 10^e^
5-F-AMT	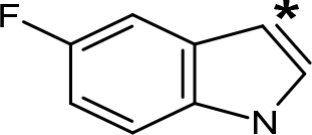	0.45^e^(0.032^f^)	376^e^(575^f^)
5-Cl-AMT	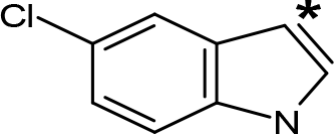	0.25^e^	82^e^
7-Me-AMT	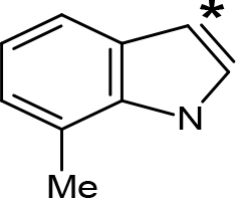	0.049^e^	> 10^e^

^a^IC_50_ and/or K_i_ are reported, depending on the reference considered. ^b^Chemical name and/or common acronym and/or common name is given. ^c^ Vilches-Herrera et al., 2009. ^d^ Vallejos et al., 2005. ^e^ Wagmann et al., 2017. ^f^ Kinemuchi et al., 1988. NT, Not tested. Ar, Aromatic ring. *: This symbol denotes where, in the aromatic ring, the aliphatic side chain is linked. Me, Methyl; Et, Ethyl; Pr, Propyl; Bu, Butyl.

Despite these considerations, some general conclusions about the MAO inhibitory activity of AMPH derivatives can be established. Thus, in the vast majority of the cases in which a relevant inhibitory activity was detected, this effect shows a clear selectivity towards MAO-A (e.g. Mantle et al., 1976; Green and el Hait, 1980; Ask et al., 1982a and Ask et al., 1982b; Ask et al., 1985; Florvall et al., 1986; Scorza et al., 1997; Gallardo-Godoy et al., 2005; Matsumoto et al., 2014; **Tables 1**–**5**). In addition, when assessed, AMPH derivatives produce in all cases a competitive and reversible^1^ inhibition of MAO (e.g. Mantle et al., 1976; Fowler and Oreland, 1981; Arai et al., 1990; Leonardi and Azmitia, 1994; Ulus et al., 2000; Fierro et al., 2007). Even though no crystal structure of MAO-A in complex with AMPH derivatives has been reported yet, docking simulations have shed light on the molecular mechanism underlying both the MAO-A inhibitory activity and the selectivity exhibited by these compounds (Vallejos et al., 2005; Fierro et al., 2007; Vilches-Herrera et al., 2009 and Vilches-Herrera et al., 2016). **Figure 2** summarizes our current view in this regard. Thus, when a substrate, in this case 5-HT, is docked at the catalytic site, it locates in a pose where the amino group is in close proximity to the isoalloxazine ring of the FAD cofactor (**Figure 2A**). This would favor the abstraction of the pro-*R* α-proton of the amine by the N5 atom of the flavin ring, which is a critical step of MAO-catalyzed amine oxidation. On the other hand, AMPH derivatives (exemplified in this case by 4-methylthioAMPH; MTA) docked at the same site, exhibit binding modes where the amino group points away from the FAD ring system (**Figure 2B**) but with the aromatic ring positioned almost identically to that of the substrate (**Figure 2C**). Indeed, such a binding mode provides a rationale for the observed inhibitory activity, since while blocking the access of any substrate to the active site, AMPH derivatives could avoid deamination by adopting a pose where the amino group is remote from the influence of the flavin ring. Furthermore, in **Figure 2D** the active site of MAO-B was superimposed on the corresponding site of MAO-A already docked with MTA. As shown, the presence of Y326 in MAO-B (I335 being the corresponding residue in MAO-A), could prevent the close fit of MTA into the active site of MAO-B. Thus, our docking experiments suggest a possible explanation for the MAO-A selectivity exhibited by most AMPH derivatives. It is worth pointing out that fragments I335 and Y326 in MAO-A and MAO-B, respectively, have been regarded as major determinants of selectivity for both substrates and inhibitors (Edmondson et al., 2007; Binda et al., 2011; Iacovino et al., 2018).

## Structure-Activity Relationships of Amph Derivatives as Maoi

### Modifications of the Side Chain

**Figure 3:**
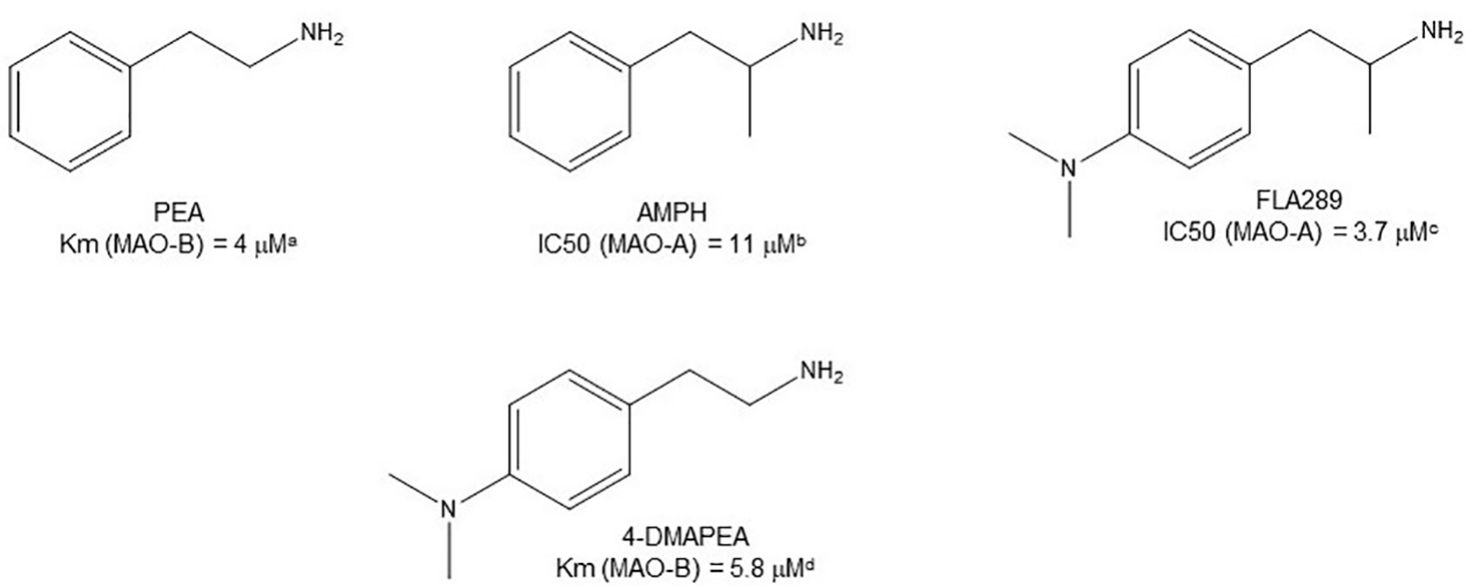
Chemical structures and MAO parameters of some phenethylamine/AMPH derivative pairs. ^a^ Youdim et al., 2006. ^b^ Scorza et al., 1997. ^c^ Reyes-Parada et al., 1994b. ^d^ Reyes-Parada et al., 1994a.

**Figure 4:**
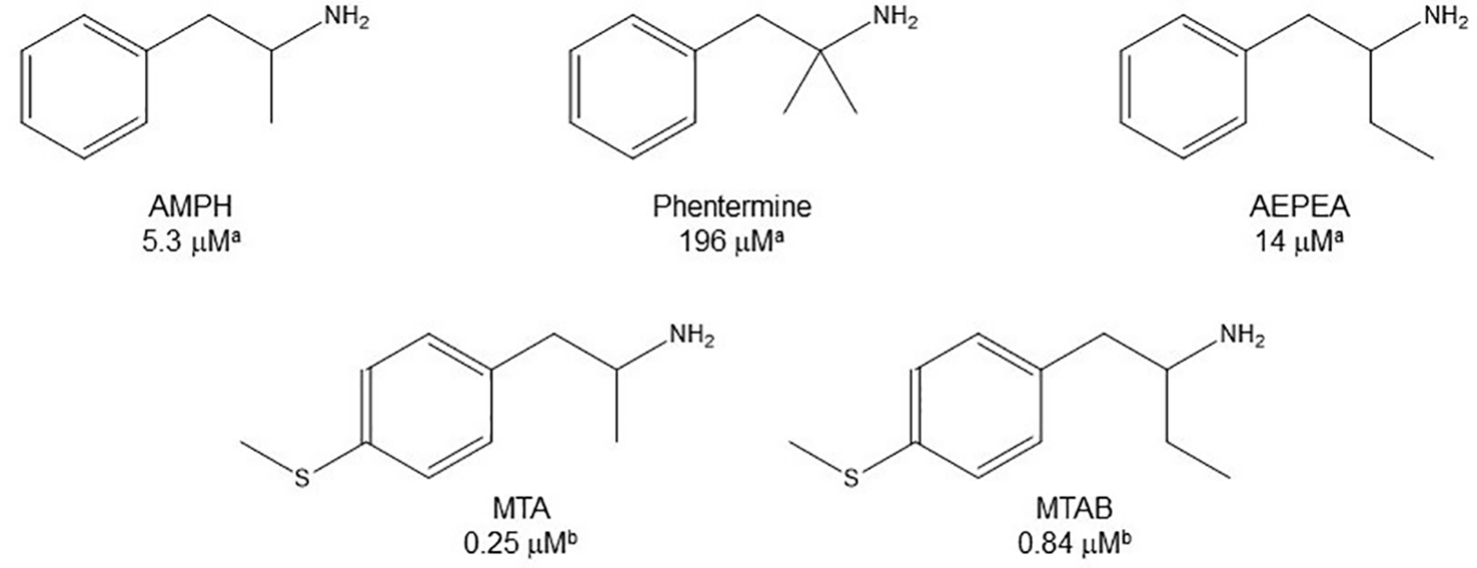
Chemical structures and MAO-A IC_50_ of some α-substituted AMPH derivatives. ^a^ Santillo, 2014. ^b^ Hurtado-Guzmán et al., 2003.

The presence of a methyl group on the α-carbon atom of phenethylamine transforms this compound, which is a selective MAO-B substrate, into AMPH which is a selective MAO-A inhibitor. This substrate-to-inhibitor change has also been reported for other phenethylamine/AMPH derivative pairs (e.g. Edwards, 1978; Yu, 1986; Fagervall et al., 1988; Reyes-Parada et al., 1994a and Reyes-Parada et al., 1994b; **Tables 1**–**2**. **Figure 3**). Considering that the α-C-H bond cleavage is likely the rate-limiting step in the catalytic cycle of both MAO isozymes (Miller and Edmondson, 1999), it seems reasonable that impeding/altering the feasibility of this step results in MAO inhibitory properties. Although stereospecific abstraction of the pro-*R*-hydrogen has been demonstrated with several substrates (Yu et al., 1986; Yu, 1988), it is noteworthy that both isomers of chiral AMPH derivatives inhibit MAOs, regardless of whether the pro-*R*- or the pro-*S*-hydrogen is the substituted atom (see **Tables 1**–**4** and references therein). This supports our idea that MAOI properties of AMPH derivatives are not necessarily due to the hindrance of hydrogen abstraction, but that the introduction of alkyl substituents at the α-carbon atom of the phenethylamine results in its adopting a different binding mode in the catalytic site (**Figure 2**). Nevertheless, extension of the alkyl substituent on the α-carbon atom or the introduction of a second methyl group at this position, lead to a decrease of MAOI-A potency compared with the parent compounds (**Figure 4**). Moreover, cyclization of the side chain to generate 2-aminoindan or 2-aminotetraline analogues results in a marked decrease of the affinity of these compounds for MAOs (Feenstra et al., 1983; Scorza et al., 1999). Such reductions of inhibitory activity would presumably reflect impediments to binding in the enzyme's active site.

**Figure 5:**
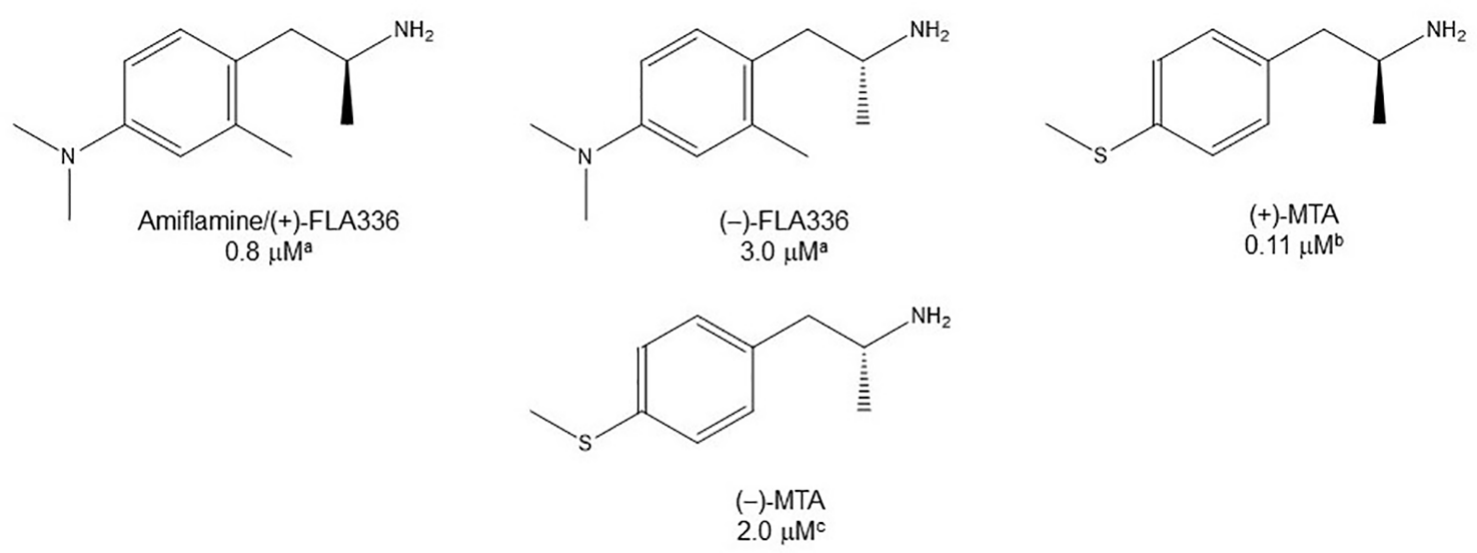
Chemical structures and MAO-A IC_50_ of some enantiomerically pure AMPH derivatives. ^a^ Ask et al., 1982b. ^b^ Fierro et al., 2007. ^c^ Hurtado-Guzmán et al., 2003.

As shown in **Tables 1**, **2**, and **4**, in most of the instances in which the dependence of enzyme inhibition on the chirality of the α-carbon atom has been tested, it has been found that the (*S*)-(+)-AMPH derivatives are the eutomers as MAOI-A (Mantle et al., 1976; Fowler and Oreland, 1981; Ask et al., 1982b; Robinson, 1985; Leonardi and Azmitia, 1994; Hurtado-Guzmán et al., 2003; **Figure 5**). As stated before, the difference in MAO-A inhibitory potency between optical isomers of AMPH derivatives is, in general, not remarkable (**Tables 1**–**4**). This suggests that stereoselectivity may not be as influential in the pharmacodynamics of these compounds as has been shown to be for the effects of AMPH derivatives upon other monoaminergic target proteins such as monoamine transporters or 5-HT receptors (Nichols, 1994; Nichols, 2018).

**Figure 6:**
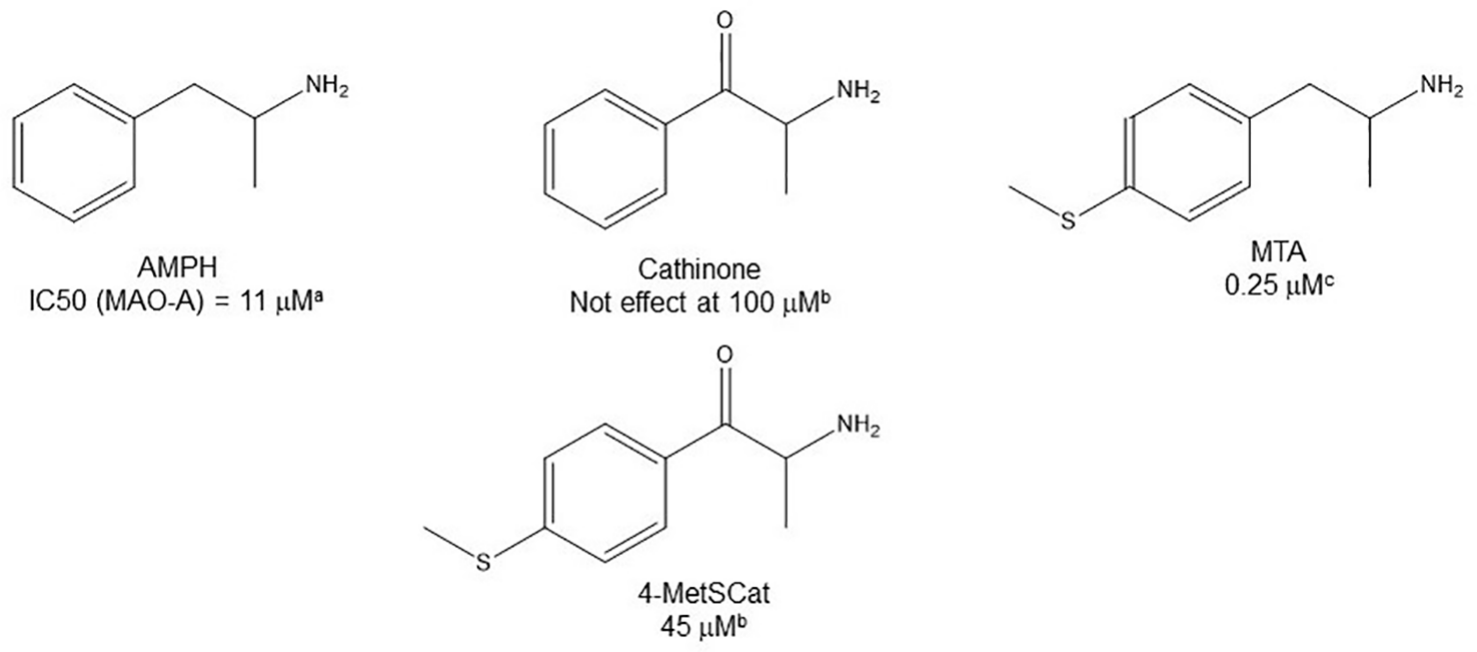
Chemical structures and MAO-A IC_50_ of some β-keto substituted AMPH derivatives. ^a^ Scorza et al., 1997. ^b^ Osorio-Olivares et al., 2004. ^c^ Hurtado-Guzmán et al., 2003.

Given the notoriety that the recreational use of cathinone derivatives has reached in the last few years (Simmler et al., 2013; Paillet-Loilier et al., 2014; Angoa-Pérez et al., 2017), it is somewhat surprising that MAOI properties of this type of compounds (as an example of β-substituted AMPH derivatives) have not been extensively studied. Nevertheless, Osorio-Olivares et al. (2004) reported that cathinone is almost completely devoid of activity as a MAOI, whereas some derivatives with alkylthio or alkoxy groups at the *para* position of the aromatic ring have IC_50_ values in the low micromolar range (**Table 3**). These results indicate that β-keto substitution of AMPH may lead to a decrease in MAOI-A potency (**Figure 6**). Nevertheless, some of these compounds showed an interesting MAO-B inhibiting activity (**Table 3**), which suggests that selectivity can be effectively modulated by side-chain substituents. In addition, β-keto substitution seems to diminish the enantioselectivity for MAO inhibition, since in some cases in which it was evaluated, the *R(-)*-derivatives were the eutomers against MAO-A. Furthermore, when the β-keto substituent was replaced by a hydroxyl group, the compounds lost their activity on MAO-B while retaining MAOI-A inhibitory properties, although with lower potencies than their AMPH counterparts (Osorio-Olivares et al., 2004; **Table 3**).

### *N*-Substitution

**Figure 7:**
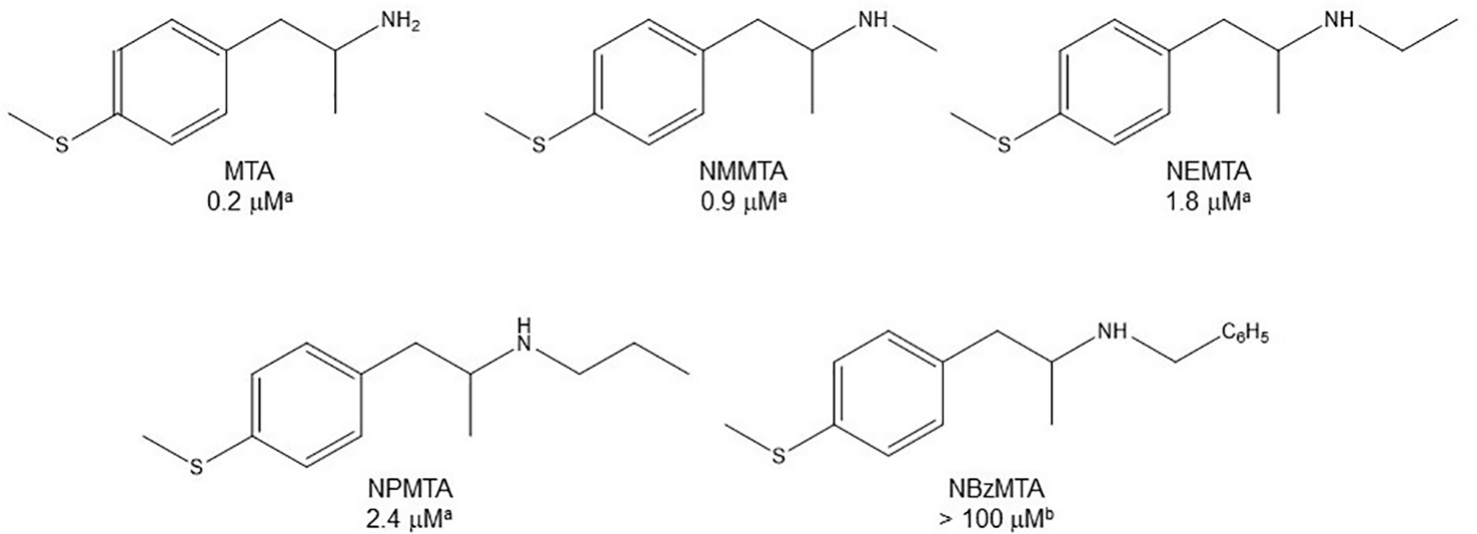
Chemical structures and MAO-A IC_50_ of some *N*-substituted AMPH derivatives. ^a^ Hurtado-Guzmán et al., 2003. ^b^ Vilches-Herrera et al., 2009.

Relatively few amino group substituents have been studied in AMPH derivatives regarding their influence upon MAOI potency. In general terms, any *N*-substitution leads to a decrease in the activity of the compound as a MAOI-A. Thus, the *N*-methyl derivatives of AMPH, MTA, *p*-methoxyAMPH (PMA), and 3,4-methylenedioxyAMPH (MDA)— i.e. methamphetamine, NMMTA, PMMA, and MDMA respectively— have about one-third the inhibitory potency of their corresponding primary amine congeners (Scorza et al., 1997; Hurtado-Guzmán et al., 2003; Matsumoto et al., 2014; Santillo, 2014; **Tables 1**, **2**, **4**). In addition, enlargement of the amine substituent to *N*-ethyl, *N*-*n*-propyl, or *N*-allyl seems to cause a further decrease in MAO-A affinity, correlated with the length of the substituent (Kilpatrick et al., 2001; Hurtado-Guzmán et al., 2003; Santillo, 2014; **Figure 7**). Even larger substituents such as *N*-benzyl can lead to a complete loss of MAOI properties (Vilches-Herrera et al., 2009). Furthermore, tertiary amines (i.e. derivatives with a second group upon the amino moiety) are even less potent as MAOI-A than their secondary or primary analogues (Hurtado-Guzmán et al., 2003). These structure-activity relationships are very similar to those observed for AMPH derivatives regarding their interactions with DAT, NET, and SERT (Nichols, 1994), which agrees with a recent report that demonstrates a remarkable structural similarity between the ligand binding sites of MAOs and monoamine transporters (Nuñez-Vivanco et al., 2018).

### Aromatic Ring Substitution

**Figure 8:**
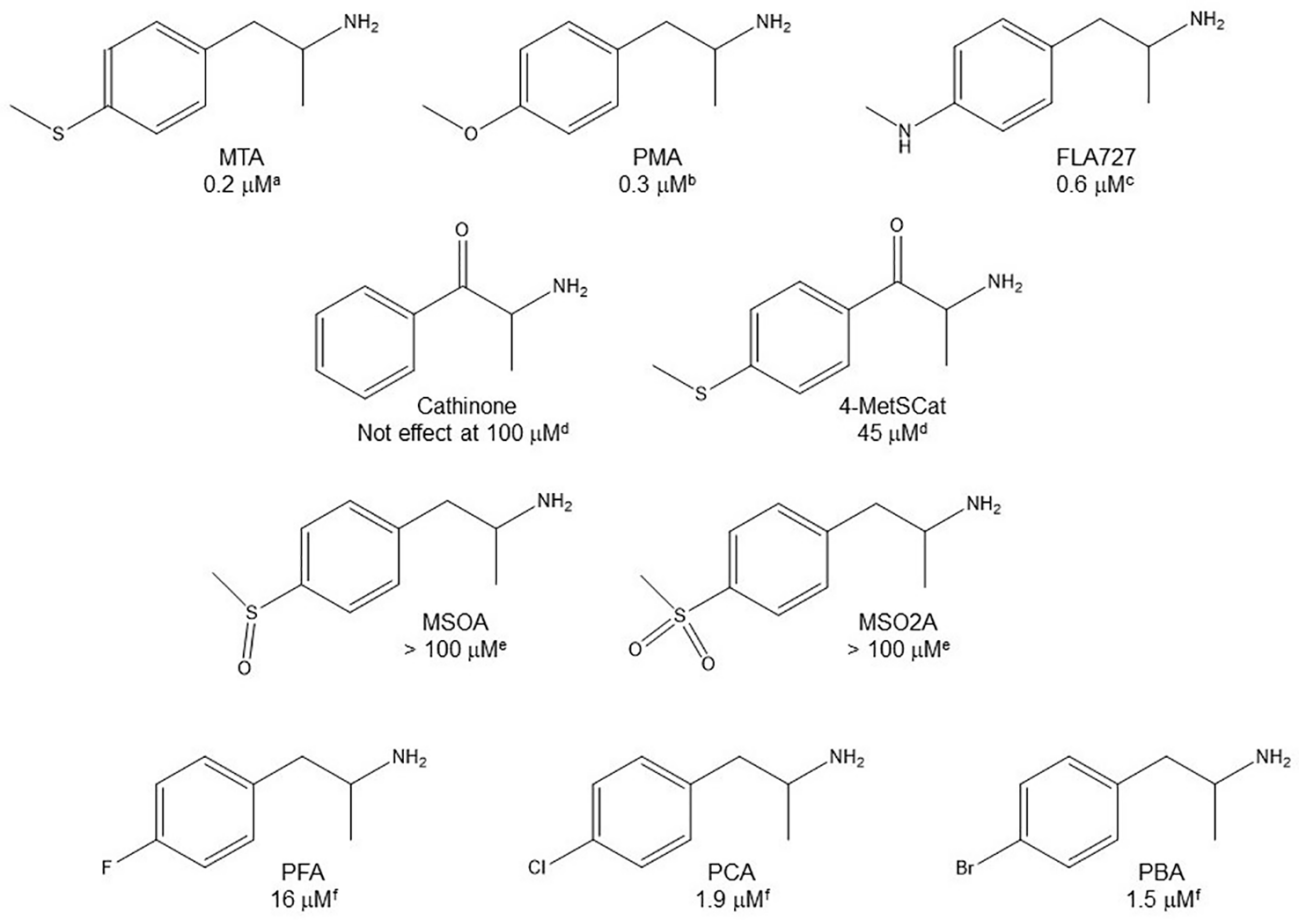
Chemical structures and MAO-A IC_50_ of some *p*-substituted AMPH derivatives. ^a^ Hurtado-Guzmán et al., 2003. ^b^ Scorza et al., 1997. ^c^ Reyes-Parada et al., 1994a. ^d^ Osorio-Olivares et al., 2004. ^e^ Vallejos et al., 2002. ^f^ Fuller et al., 1975.

**Figure 9:**
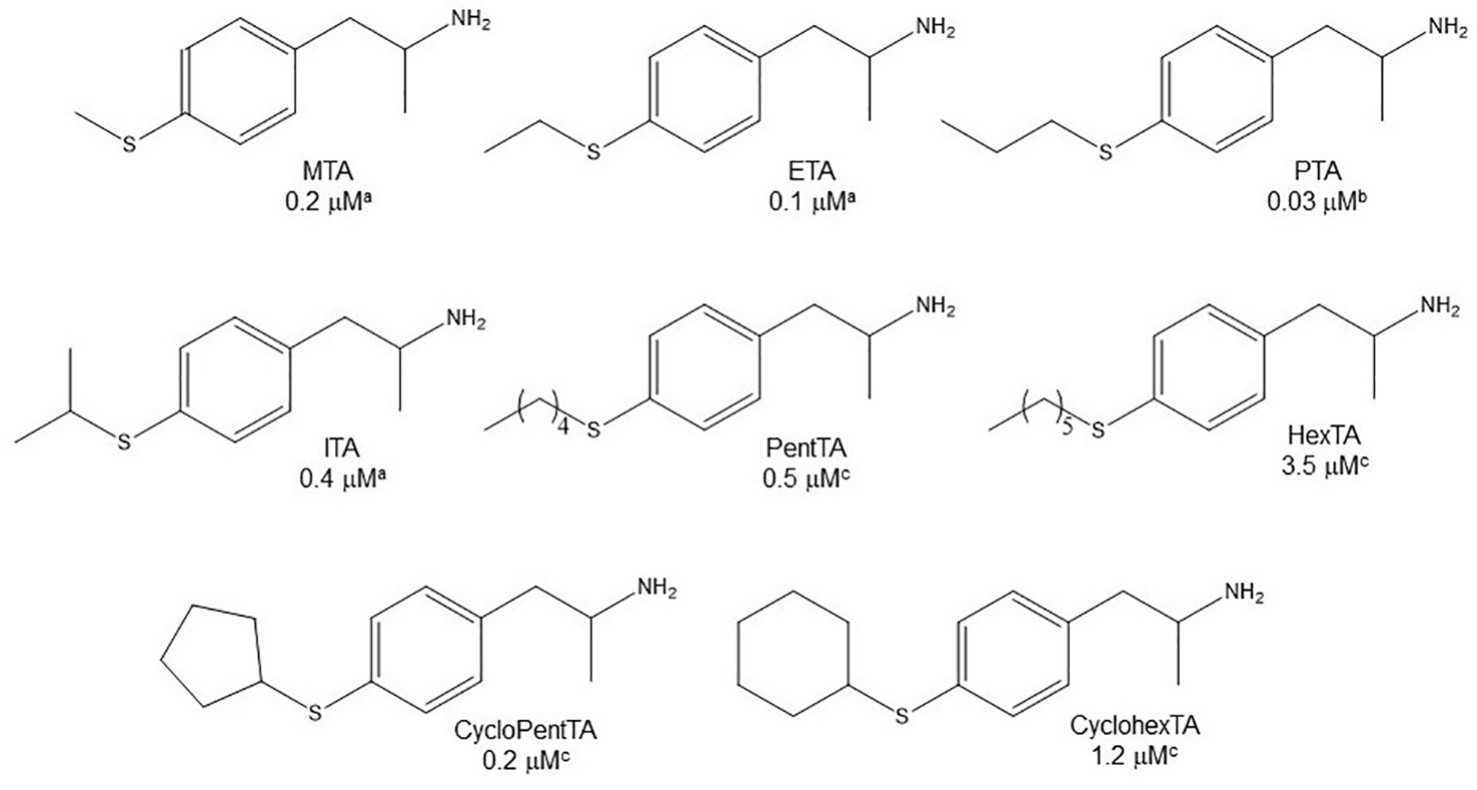
Chemical structures and MAO-A IC_50_ of some *p*-alkylthio AMPH derivatives. ^a^ Scorza et al., 1997. ^b^ Fierro et al., 2007. ^cVilches-Herrera et al., 2016^.

**Figure 10:**
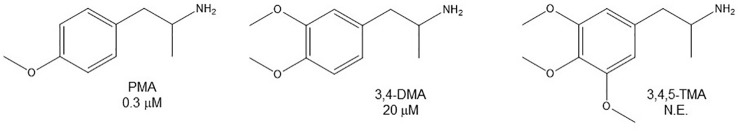
Chemical structures and MAO-A IC_50_ of some *p*-methoxy AMPH derivatives. IC_50_ values are from Scorza et al., 1997. NE, No effect.

Even though some aliphatic MAO substrates and inhibitors have been identified (Yu et al., 1994; Yu et al., 1995; Kalgutkar et al., 2001), most studies have shown that the presence of an aromatic ring is essential for potent MAOI activity (Wouters, 1998; Kalgutkar et al., 2001; Tripathi et al., 2018; Dhiman et al., 2018). In the specific case of AMPH derivatives, docking simulations indicate that the benzene ring of these compounds binds to MAO-A mainly *via* interactions with π-systems of the catalytic site residues such as Y407, Y444 (both of which form part of the so called “aromatic cage”), Y69, F208, and/or F352 (Vallejos et al., 2005; Fierro et al., 2007; Vilches-Herrera et al., 2009; Fresqui et al., 2013; Vilches-Herrera et al., 2016; **Figure 2**). Accordingly, a handful of QSAR studies have shown that electronic features of the benzene ring (e.g. CHELPG atomic charges, HOMO energies) are the most important factors to determine the affinity of AMPH derivatives for MAO-A, and hence that their variation caused by substituents can greatly modulate their potency as enzyme inhibitors (Norinder et al, 1994; Vallejos et al., 2002; Fresqui et al., 2013). In this respect, the presence of a single substituent with electron-donor properties (e.g. alkoxy, alkylthio, alkylamino) at the *para* position of the aromatic ring seems to be the most influential substitution favoring potency toward MAO-A. Thus, MTA, PMA, and *p*-methylaminoAMPH (FLA727) are 20-50-fold more potent than the parent compound (Ask et al., 1985; Scorza et al., 1997; **Tables 1**, **2**, **Figure 8**). The importance of the electron-donor character of the substituent at the *para* position is substantiated by the significant decrease of potency observed when the *p*-methylthio group is replaced by electron-withdrawing moieties such as *p*-methylsulfoxy or *p*-methylsulfonyl (i.e. MSOA or MSO2A, respectively; Vallejos et al., 2002; **Table 2**, **Figure 8**). It is worth pointing out that *para*-halogenated AMPHs (PCA, PBA, and PFA) are also fairly potent MAOI-A, with their potencies negatively correlated with their electron-withdrawing character, that is, PBA and PCA being about 7- and 4-fold more potent, and PFA being slightly less potent than AMPH (Fuller et al., 1975; Scorza et al., 1997; **Table 2**, **Figure 8**). Modestly increasing the size of the *para*-sulfur/oxygen substituent with linear aliphatic chains (i.e. ethyl-, propyl-, butyl-) leads to an increase in potency, while larger or branched substituents generate less potent compounds (Scorza et al., 1997; Gallardo-Godoy et al., 2005; Fierro et al., 2007; Vilches-Herrera et al., 2009 and Vilches-Herrera et al., 2016; **Figure 9**). It has been suggested (Fierro et al., 2007) that the increase in potency might be related to steric parameters of the *para* substituent (van der Waals volume and/or Taft steric parameter *E_s_*) which would be optimal for alkyl chains containing up to four carbon atoms. Longer chains, however, might oblige the compound to adopt a more folded conformation, which would disfavor their interaction with the residues at the binding site. Interestingly, cycloalkyl analogues of pentylthioAMPH and hexylthioAMPH showed higher rat MAOI-A potencies than their *n*-alkyl counterparts, which was attributed to a better fit within the binding site due to the entropic advantage conferred by their “precoiled” conformations (Vilches-Herrera et al., 2016; **Figure 9**). In contrast, the presence of substituents at the *ortho* or *meta* positions in *para*-unsubstituted compounds had no effect or led to a decrease of the potency as MAOI-A as compared with AMPH (Green and el Hait, 1980; Scorza et al., 1997; Kilpatrick et al., 2001; **Tables 2** and **4**). Moreover, the addition of bulky groups adjacent to the *para*-substituted position induces a decrease in potency. Thus, the presence of one or two substituents at the *meta* position(s) of PMA produce compounds that are respectively 60-fold less potent or completely inactive (Scorza et al., 1997; **Figure 10**). Nonetheless, the introduction of substituents at more distant positions has less detrimental effects on potency, and even in some cases a marked increase of activity (and selectivity) has been reported. Thus, the addition of a methyl group at the *ortho* position of *p*-dimethylaminoAMPH (FLA289) to yield FLA336 produced a slight increase in potency, which was more evident (along with a remarkable increase in MAO-A/B selectivity) in the case of amiflamine, the (*S*)-(+)-isomer of FLA336 (Ask et al., 1985; **Table 1**). Indeed, the introduction of one or two halogen atoms (Cl or Br) at the *ortho* position(s), generates some of the most potent MAOI-A AMPH derivatives described until now (Ask et al., 1985; Gallardo-Godoy et al., 2005; **Tables 1** and **4**).

The expansion of the aromatic ring of AMPH is an additional modification that has yielded interesting results and potent and selective MAOIs-A (**Table 5**). This could have been anticipated considering that: a) the structure of 5-HT, one of the natural substrates of MAO-A, contains an indolyl moiety instead a simple benzene ring and; b) the electron-richness of the aromatic ring seems to be an important molecular determinant for MAO-A affinity (Vallejos et al., 2002), and therefore aromatic systems larger than benzene might establish stronger π-type interactions with aromatic and non-aromatic residues present in the active site of the protein. Accordingly, for example 2-naphthylisopropylamine (NIPA, also known as PAL-287; Rothman et al., 2005) and its methoxylated or methylthio derivatives (Vilches-Herrera et al., 2009), 2-benzofurylisopropylamine (Vallejos et al., 2005) or several α-methyltryptamine derivatives (Kinemuchi et al., 1988; Wagmann et al., 2017) have been shown to be highly selective MAOI-A, much more potent than AMPH (**Table 5**). Both electronic and steric factors have been invoked to explain the higher activity of AMPH derivatives containing aromatic rings larger than benzene (Vallejos et al., 2005; Vilches-Herrera et al., 2009). Thus, π systems with an increased electron-donating capacity, softer and/or more polarizable as compared with benzene, might favor charge-transfer (Vallejos et al., 2002) and/or π-stacking interactions (Fresqui et al., 2013) with aromatic fragments in the active site. In addition, docking simulations have shown that AMPH derivatives containing large aromatic rings, can establish interactions not only with the aromatic residues forming the so-called aromatic cage (i.e. Y407 and Y444), but also with aminoacids located more distantly, such as F208, Y69, and F352 (Vallejos et al., 2005; Vilches-Herrera et al., 2009). Therefore, the higher potency of AMPH derivatives containing condensed aromatic systems could be explained by an increased probability of establishing dispersive short length interactions and also a greater number of interactions.

## Summary of Structure-Activity Relationships and Implications of Maoi Properties for the Overall Pharmacology of Amph Derivatives

Potent, selective, and competitive MAO-A inhibitory properties are found in many AMPH derivatives. This is likely due to the structural similarity of this type of compounds with physiological substrates, which allows AMPH derivatives to occupy, and consequently block the access of any substrate into the active site of the enzyme.

Although not extensively studied, in general the introduction of diverse substituents on either the amino group or the side chain of the basic AMPH skeleton leads to compounds with lower affinity as compared with the parent counterparts. Furthermore, several studies have shown that (*S*)-(+)-AMPH derivatives are the eutomers for MAO inhibition. Besides, as aromatic interactions in the active site of the enzyme seem to be critical for inhibition, substituents at this portion of the AMPH structure greatly modulate its potency. In general, electron-donor substituents at the *para* position of the aromatic ring generate potent MAOI-A, while substituents adjacent to this position decrease activity. In addition, replacement of the benzene ring by larger π systems exhibiting an increased electron-donating capacity, generates compounds with higher MAOI potency.

Even though MAO inhibition has been demonstrated for several AMPH derivatives, this is often considered not relevant for their global effect, since their affinity for MAO is usually weak compared to affinity for their main pharmacological targets (i.e. monoamine transporters or receptors). This is most likely true in the case of compounds such as the anti-obesity agents phentermine and fenfluramine (Kilpatrick et al., 2001; Nandigama et al., 2002), or the hallucinogenic drugs DOI and DOB (Nichols, 2018), whose ability either to evoke monoamine release or to activate 5-HT receptors exceeds by several orders of magnitude their potency as MAOIs. However, in the case of monoamine releasing agents such as PMA or MTA, their potency upon their main protein targets (i.e. SERT and DAT) is remarkably similar to that reported for MAO-A (Green and el Hait, 1980; Nichols et al., 1993; Scorza et al., 1997; Callaghan et al., 2005; Gobbi et al., 2008; Sotomayor-Zárate et al., 2012; Matsumoto et al., 2014). Thus, one may assume that the cases of severe toxicity reported after recreational use of these drugs (e.g. Elliott, 2000; De Letter et al., 2001; Martin, 2001; Lamberth et al., 2008), which resemble “serotonin syndrome” symptoms (Lapoint et al., 2013), are related to a sustained increase of synaptic 5-HT and DA resulting from both monoamine reverse transport and MAO-A inhibition. Furthermore, as many AMPH derivatives are monoamine transporter substrates (Simmler et al., 2013; Sitte and Freissmuth, 2015), even in those cases in which relatively weak MAOI activity is demonstrated, these drugs might be concentrated in presynaptic nerve terminals or glial cells, and some enzyme inhibition could occur (Heal et al., 2013). In this sense, accumulation and pronounced inhibition of MAO in monoaminergic neurons has been consistently reported for amiflamine and its analogues (Ask et al., 1983; Ask et al., 1985; Ask et al., 1986; Ask and Ross, 1987). In addition, especially after relatively prolonged use, antidepressant and/or anxiolytic effects derived from MAO-A inhibition might influence the global pharmacological effect of AMPH derivatives. Thus, for example, considering the high overlap between depression and drug abuse (Markou et al., 1998; Bruijnzeel et al., 2004), it is enticing to suggest that at least part of the antiaddictive potential of PAL-287 (NIPA; **Table 5**), which has been attributed to its DA and 5-HT releasing properties (Rothman et al., 2005; Rothman et al., 2007; Rothman et al., 2008), might be related also to its MAOI-A activity (Vilches-Herrera et al., 2009). Moreover, subtle differences in the subjective experience generated by hallucinogenic AMPH derivatives such as DOI or DOB as compared with sulfur containing analogues (Shulgin and Shulgin, 1991), might be associated with the much more pronounced MAOI-A activity of the latter (**Table 4**, Scorza et al., 1997; Gallardo-Godoy et al., 2005). Thus, these and other examples highlight the notion that MAOI properties should be considered when assessing the overall pharmacology of AMPH derivatives.

## Concluding Remarks

Although many AMPH derivatives have been tested as MAOI, the structural diversity of such compounds is relatively limited. This calls for a broader exploration of the chemical space around the parent scaffold in the search of compounds with novel properties, in which MAOI properties might or might not be pursued. As known and unknown AMPH derivatives are usually attractive for illicit purposes (production, marketing, and/or consumption), it seems very relevant to evaluate in every case the possible MAOI activity of these drugs, since it may convey dangerous consequences for uninformed users.

Regarding the mechanism of enzyme inhibition, insofar as a crystal structure of MAO in complex with some AMPH derivative is not available, molecular simulation appears as one of the most reliable tools to study this issue. Nevertheless, most of current information has been obtained through docking studies, without resorting to molecular dynamics simulations that consume much more computer time, and therefore models generated still require a further validation. In addition, several reports indicate that MAOI profiles differ if enzymes from human or other species are used (not only for AMPH derivatives). Hence, inferences regarding possible effects in humans should be most cautious when data are obtained initially in animal models. Moreover, in comparison to recent characterizations of the monoamine transporter and receptor interactions of amphetamines (Simmler et al., 2013; Luethi and Liechti, 2018), the MAO inhibiting properties have not been investigated using the same assays across a larger range of substances. Therefore, comparative analyses should be done cautiously when considering results obtained under different experimental conditions

Beyond these considerations, in our view it is clear that AMPH derivatives can act as MAOI and that this activity should be taken into account when analyzing the overall pharmacodynamics of these structurally versatile compounds.

## Author Contributions

All authors equally contributed to the writing of this manuscript.

## Funding

The constant support of FONDECYT, in particular grants 1170662 (MR-P), 1150615 (PI-V), and 1150868 (BKC), is gratefully acknowledged.

## Conflict of Interest

The authors declare that the research was conducted in the absence of any commercial or financial relationships that could be construed as a potential conflict of interest.

## Footnotes

1 It should be noted that we do not review here irreversible inhibitors such as deprenyl or tranylcypromine. These compounds and their analogues can also be considered as AMPH derivatives, but due to particular structural characteristics (the presence either of a propargyl moiety on the amino group or a cyclopropyl ring involving the α and β carbons of the side chain, respectively), they are suicide substrates that can form covalent adducts with the flavin ring of the enzyme's cofactor. This characteristic leads to an irreversible mode of inhibition and generates completely different structure-activity relationships. These have been analyzed elsewhere (e.g. Magyar, 1994; Yoshida et al., 2004; Malcomson et al., 2015) and the reader is also referred to the literature (Binda et al., 2003; De Colibus et al., 2005) to have insights regarding the actual structural characteristics of the MAO-drug adducts formed by these compounds.

## Abbreviations

AMPH, amphetamine; DA, dopamine; NE, norepinephrine; DAT, dopamine transporter; NET, norepinephrine transporter; 5-HT, serotonin; SERT, serotonin transporter; MAO, monoamine oxidase; FAD, flavin adenine dinucleotide; MAOI, monoamine oxidase inhibitor; MTA, 4-methylthioamphetamine; PMA, *p*-methoxyamphetamine; MDA, 3,4-methylenedioxyamphetamine; NMMTA, 4-methylthiomethamphetamine; PMMA, *p*-methoxymethamphetamine; MDMA, 3,4-methylenedioxymethamphetamine; FLA727, *p*-methylaminoamphetamine; MSOA, *p*-methylsulfoxyamphetamine; MSO2A, *p*-methylsulfonylamphetamine; PCA, *p*-chloroamphetamine; PBA, *p*-bromoamphetamine; PFA, *p*-fluoroamphetamine; FLA289, *p*-dimethylaminoamphetamine; NIPA, 2-naphthylisopropylamine (also known as PAL-287); DOI, 4-iodo-2,5,-dimethoxyamphetamine; DOB, 4-bromo-2,5,-dimethoxyamphetamine.
